# ABA and stomatal biology: from transcriptional and biosynthesis to multicellular coordination across cell types

**DOI:** 10.1093/plphys/kiag773

**Published:** 2026-10-19

**Authors:** Bo Xu, Zhong-Hua Chen

**Affiliations:** ARC Centre of Excellence in Plants for Space, and School of Agriculture, Food and Wine, Waite Research Institute, Adelaide University, Glen Osmond, SA 5064, Australia; School of Agriculture, Food and Wine, Waite Research Institute, University of Adelaide, Glen Osmond, SA 5064, Australia; School of Agriculture, Food and Wine, Waite Research Institute, University of Adelaide, Glen Osmond, SA 5064, Australia

## Abstract

Abscisic acid (ABA) was first identified in plants over 6 decades ago and is now recognized as one of the central phytohormones in plants, regulating growth, development, and physiological adaptation to environmental changes. In this Centennial Update, we use two influential ABA-related articles published in *Plant Physiolog*y in the early 2000s and discuss complementary advances in stress-responsive transcription and ABA biosynthetic regulation. We extend the Update from ABA-relevant gene discovery to recent advances in multicellular coordination of stomatal control. Growing evidence supports the view that ABA-regulated stomatal movement and gas exchange are coordinated across multiple cell types, including guard cells, mesophyll cells, bundle sheath cells, and vascular tissues in roots and leaves. We also highlight emerging tools to dissect this ABA-mediated multicellular network by using emerging technologies in single-cell and spatial omics, plant optogenetics, and artificial intelligence. These advances will shape ABA research from single cell models toward multicellular collaborative networks for understanding plant-environment interactions. Together, this article links the history of ABA research with emerging single-cell resolution datasets and artificial intelligence–assisted computational approaches, proposing a framework for moving ABA biology from well-established guard cell signaling models toward a broader understanding of multicellular ABA coordination.

## Introduction

Abscisic acid (ABA) is one of the central phytohormones, coupling developmental programs with responses to environmental change in plants. ABA-mediated metabolic and signaling pathways are important for a broad range of plant biological processes, including seed dormancy, germination, flowering, ion and solute transport, and epigenetic regulation, as well as biotic and abiotic stress adaptation (Chang et al 2020; Chen et al 2020; Kishor et al 2022). Mechanistically, many key elements of ABA biosynthesis, catabolism, transport, perception, and downstream signaling have been identified through biochemical, genetic, molecular, and physiological approaches. These discoveries have provided a framework for engineering ABA signaling pathways to improve plant stress resilience, particularly under water-scarcity conditions in which ABA regulates transpirational water loss, water-use efficiency, and survival. Many aspects have been extensively discussed in recent reviews (Lawson and Matthews 2020; Pantoja 2021; Waadt et al 2022; da Silva et al 2025). Therefore, this Update does not aim to comprehensively cover all facets of plant ABA biology. Instead, it focuses on ABA regulated stomatal behavior and gas exchange, particularly highlighting how ABA biosynthesis, transport, and signaling are coordinated across multiple cell types, while guard cells remain the major effectors of stomatal movement.

We first revisit the history of plant ABA research in the context of two influential *Plant Physiology* articles—Rabbani et al (2003) and Xiong and Zhu (2003)—by discussing how later work extended these foundations from transcriptional and metabolic regulation to multicellular coordination of ABA signaling. Among these processes, guard cells serve as a central signaling hub in plants for ABA-mediated stomatal movement and gas exchange—a process that enables carbon dioxide (CO_2_) uptake for photosynthesis and release of oxygen (O_2_) and water (H_2_O) via transpiration (Lawson and Matthews 2020; Kishor et al 2022). Guard cells, typically paired kidney shape cells surrounding stomatal pore in eudicots, adjust stomatal pore size and have provided a core framework for understanding ABA signaling (Hsu et al 2021). However, stomatal function is also shaped by other leaf and vascular cell types, which influence guard cells by regulating leaf water supply (Scharwies and Dinneny 2019), photosynthesis-derived metabolic signals (Fujita et al 2013; Zait et al 2025), and root-to-shoot long-distance hydraulic, hormonal, and peptide signals (Takahashi et al 2018; Zhang et al 2021). Consequently, stomatal regulation provides an exceptional example to understand how ABA can regulate physiological responses beyond a single cell type. We propose that single-cell and spatial omics, plant optogenetics, and artificial intelligence (AI)–assisted analysis will empower the research to dissect how ABA's mode of action is organized across multiple cell types. These toolkits may be adapted to investigate broader ABA-mediated physiological and stress-responsive processes across tissues and organs in plants.

## Advances of ABA research in *Plant Physiology*

ABA was first isolated from plants over 6 decades ago, as DL-abscisin II, initially described as a substance accelerating cotton leaf abscission (Ohkuma et al 1963). *Plant Physiology* became an early venue of ABA research in 1966, with one of the first studies published showing that DL-abscisin II inhibits the germination of excised embryos from two Ash species, *Fraxinus ornus* and *F. americana*. Such inhibitory effect could be antagonized by another phytohormone, gibberellin acid (Sondheimer and Galson 1966). Abscisin II (or dormin) was also found to stimulate flowering development in short-day plant species, such as *Chenopodium rubrum* and *Pharbitis nil* (El-Antably and Wareing 1966) and reduce transpiration in excised leaves of wheat (*Triticum aestivum*) and barley (*Hordeum vulgare*) (Mittelheuser and Van Steveninck 1969). This compound was formally renamed as abscisic acid, to reconcile the parallel names abscisin II and dormin (Addicott et al 1968). By the early 2000s, major advances had been made in understanding ABA biology in plants, including ABA-mediated ion transport in guard cells, signaling crosstalk (Fedoroff 2002), transcriptional responses (Rock 2000), and the identification of core ABA biosynthetic enzymes (Seo and Koshiba 2002). However, less was known about how such biosynthesis was regulated by developmental and environmental cues, and how ABA-mediated transcriptional networks were conserved or diversified beyond *Arabidopsis thaliana*.

*Plant Physiology* has been the major venue for plant ABA research, with >1,500 ABA-related articles published (Web of Science 2026). Within this broader history, we revisit two influential *Plant Physiology* articles that provide complementary perspectives on ABA-responsive transcription (Rabbani et al 2003) and ABA biosynthesis and regulation (Xiong and Zhu 2003). These studies built on substantial earlier advances in ABA and stress biology. For instance, Kreps et al (2002) had investigated global expression profiles in Arabidopsis to probe shared and stress-specific transcriptional response to cold, salt, and osmotic stress, while Finkelstein et al (2002) integrated early genetic and biochemical evidence into a conceptual framework for ABA signaling. Rabbani et al (2003) extended this transcriptomic framework to rice (*Oryza sativa*) and included ABA as a separated treatment, alongside cold, drought, and high salinity. Using a microarray comprising ∼1,700 complementary DNAs with RNA gel-blot analyses, the authors reported >70 stress-inducible genes, including >50 novel candidates not previously described in rice. Intriguingly, more than half identified rice orthologous genes had been reported in *Arabidopsis*, suggesting that *Arabidopsis* and rice may share conserved ABA regulatory mechanism for plant abiotic stress adaptation. Notably, 15 rice genes induced by all 4 treatments underscored shared core ABA-responsive components across stress contexts (Rabbani et al 2003).

Among these ABA-responsive genes identified by Rabbani et al (2003) were ABA-biosynthetic genes (e.g. OsZEP/ABA1 and OsSDR/ABA2), providing a natural link to a conceptual updated by Xiong and Zhu (2003) on regulation of ABA biosynthesis. Xiong and Zhu (2003) synthesized emerging evidence that ABA production is developmentally and environmentally regulated, including seed maturation, germination, and plant responses to abiotic stress. They highlighted major biosynthetic enzymes functionally characterized in Arabidopsis, including key ABA-biosynthetic marker genes—*NCED*s (Fig. 1). These enzymes form the canonical ZEP-dependent ABA biosynthetic pathway. More recent studies identified an alternative ZEP-independent ABA synthetic pathway, involving unresolved isomerization, hydroxylation and epoxidation steps (Jia et al 2022) (Fig. 1). However, evidence for this ZEP-independent pathway as a major abiotic stress-regulated ABA source remains limited (Gupta et al 2022). ABA homeostasis has also extended by the identification of oxidative catabolism through cytochrome P450 superfamily members such as CYP707As (Saito et al 2004; Okamoto et al 2011), and ABA conjugated pathway through ABA UDP-glucosyltransferases (Dong et al 2014; Liu et al 2015) (Fig. 1). A central insight from Xiong and Zhu (2003) is that ABA biosynthesis can be feedback regulated: exogenous ABA and/or stress-triggered endogenous ABA can induce transcription of ABA biosynthetic genes, reinforcing ABA accumulation under stress (Xiong and Zhu 2003). A prominent example are *NCED*s, encoding a rate-limiting enzyme for catalyzing stress-induced ABA synthesis (Thompson et al 2000). In *Arabidopsis*, *NCED3* is strongly induced by ABA and abiotic stresses such as salt, drought, and osmotic stress (Cheng et al 2002; Xiong et al 2002). Together, these features make *NCED3* a widely used reporter gene for ABA and stress-responsive ABA biosynthesis (Xiong and Zhu 2003). In the recent two decades, *NCED* expression has been repeatedly leveraged to dissect how ABA biosynthesis is modulated by diverse biotic and abiotic stresses and how this regulation contributes to stress adaptation (Chen et al 2020; Kishor et al 2022; Waadt et al 2022). Meanwhile, major advances further established the molecular framework for ABA dependent signaling, particularly in stomatal regulation, including identification of SLAC1 (Negi et al 2008; Vahisalu et al 2008), reconstitution of core ABA signaling modules (Fujii et al 2009; Brandt et al 2012), and identification of ABA transporters (Kang et al 2010; Kuromori et al 2010). These discoveries form the basis of many of the stomatal regulation and intercellular signaling mechanisms discussed subsequently.

**Figure 1:**
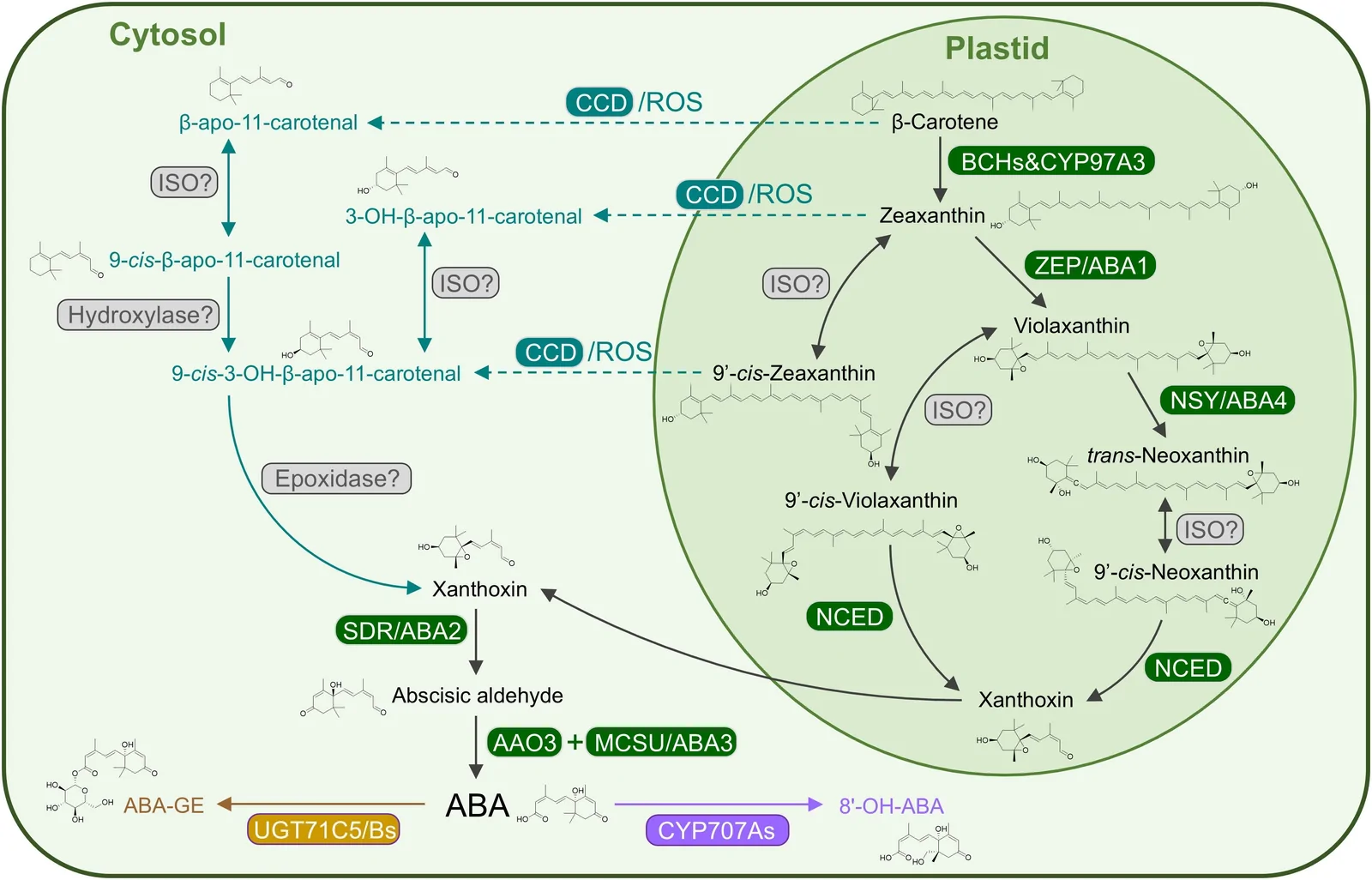
Schematic diagram of ABA biosynthesis, catabolism, and conjugation pathways in plants. ABA is synthesized from β-carotene (carotenoid precursors) in a canonical ZEP-dependent pathway (black) and through a proposed ZEP-independent (blue) pathway (Ma et al. 2018; Jia et al. 2022). In the ZEP-independent pathway (black), β-carotene is hydroxylated by nonheme di-iron carotene hydroxylases (BCHs) and heme-containing cytochrome P450-type carotene hydroxylase (CYP97A3) to produce zeaxanthin (Kim et al. 2009). Zeaxanthin is then epoxidized by ZEP (ZEP/ABA1) to generate violaxanthin (Schwartz et al. 2003), which can be further converted to trans-neoxanthin by neoxanthin synthase (NSY/ABA4) (North et al. 2007). Violaxanthin and trans-neoxanthin are isomerized by unidentified isomerase (ISO?) respectively to produce 9′-cis-violaxanthin and 9′-cis-neoxanthin (Jia et al. 2022). These 9-cis-epoxycarotenoids are then cleaved by NCEDs to produce xanthoxin, a key ABA precursor. In the ZEP-independent pathway (green), β-carotene– and zeaxanthin-derived 9-cis-3-OH-β-apo-11-carotenoids are generated through enzymatic cleavage—potentially by carotenoid cleavage dioxygenase (CCD) or NCED—and/or nonenzymatic oxidation mediated by ROS. These intermediates, including β-apo-11-carotenal, 9-cis-β-apo-11-carotenal, 3-OH-β-apo-11-carotenal, and 9-cis-3-OH-β-apo-11-carotenal, are further converted to xanthoxin by unknown hydroxylation and epoxidation steps (Jia et al. 2022). Xanthoxin is then catalyzed in the cytosol into abscisic aldehyde by xanthoxin dehydrogenase (SDR/ABA2), which is subsequently oxidized to ABA by ABA aldehyde oxidase 3 (AAO3), a reaction that requires a sulfurated molybdenum cofactor supplied by molybdenum cofactor sulfurase (MCSU/ABA3) (Schwartz et al. 2003). ABA can be oxidatively inactivated by cytochrome P450 ABA 8′-hydroxylases (CYP707As) to 8′-hydroxy-ABA (8′-OH-ABA) (purple) (Saito et al. 2004; Okamoto et al. 2011); alternatively, ABA can also be conjugated to ABA-glucose ester (ABA-GE) by ABA UDP-glucosyltransferases, including UGT71C5 and UGT71B family members (UGT71C5/Bs), as reversible storage (yellow) (Dong et al. 2014; Liu et al. 2015). Chemical structures were generated by ChemDraw 2023 based on chemical compound information available on PubChem (https://pubchem.ncbi.nlm.nih.gov/). Adapted from Xiong and Zhu (2003) and Schwartz et al. (2003), combined with information from Hauser et al. (2011); Ma et al. (2018), and Jia et al. (2022).

## Guard cell ABA signaling provides the core framework for stomatal regulation

ABA is a core regulator of guard cell function, stomatal development, and water loss across diverse environmental contexts, promoting stomatal closure under elevated CO_2_ (Hsu et al 2018), low humidity (Bauer et al 2013), and soil water deficiency (Takahashi et al 2018), while also controlling the extent of stomatal opening under light (Yin et al 2013) and high temperature (Xu et al 2025) and negatively regulating stomatal density (Jalakas et al 2024). Compared with bulk-tissue ABA-responsive studies (Rabbani et al 2003), *Arabidopsis* guard cell protoplast transcriptomics enabled cell type–specific advances, identifying >1,300 guard cell–enriched transcripts (Leonhardt et al 2004) to define ion transport, calcium (Ca^2+^) signaling, and water movement during ABA-mediated stomatal closure in *Arabidopsis*, as summarized in Chen et al (2020) and Hsu et al (2021).

Briefly, ABA in guard cells can be synthesized locally or taken up from the apoplast through the plasma membrane ABA importer ABCG40 (Kang et al 2010; Merilo et al 2015) (Fig. 2). ABA then promotes stomatal closure through interconnected Ca^2+^-dependent and Ca^2+^-independent pathways (Fig. 2). ABA-bound PYR/PYL/RCAR forms a protein complex with clade A PP2C proteins (e.g. ABI1 and ABI2), releasing OST1 from PP2C-mediated inhibition (Nishimura et al 2009; Park et al 2009; Umezawa et al 2009; Vlad et al 2009; Brandt et al 2012). Then, OST1 amplified ABA signaling through activation of interconnected reactive oxygen species (ROS)-Ca^2+^ branches. It stimulates NADPH-dependent ROS production in guard cell apoplast via activating RbohD and RbohF (Kwak et al 2003; Sirichandra et al 2009). ROS, particularly hydrogen peroxide (H_2_O_2_), can enter guard cells through OST1-dependent activation of plasma membrane aquaporin PIP2;1 (Ronzier et al 2014; Grondin et al 2015). Apoplastic H_2_O_2_ also triggers cytosolic Ca^2+^ signaling (Pei et al 2000). This ABA-dependent ROS-Ca^2+^ branch can suppress plasma membrane proton (H^+^)-ATPase (AHA) activity by promoting their dephosphorylation and reducing H^+^ extrusion (Kinoshita et al 1995; Zhang et al 2004). In addition, vacuolar H^+^-ATPase (VHA)–dependent vacuolar acidification also contributes to ABA-induced stomatal closure in guard cells (Bak et al 2013) and may be coupled with ABA-dependent Ca^2+^ signaling, as observed in bundle sheath cells (Torne-Srivastava et al 2024). Together, these processes favor cytosolic alkalinization and reduce the hyperpolarizing effect of AHA-mediated H^+^ pumping on plasma membrane potential (*E*_m_) (Zhang et al 2004; Bak et al 2013).

**Figure 2:**
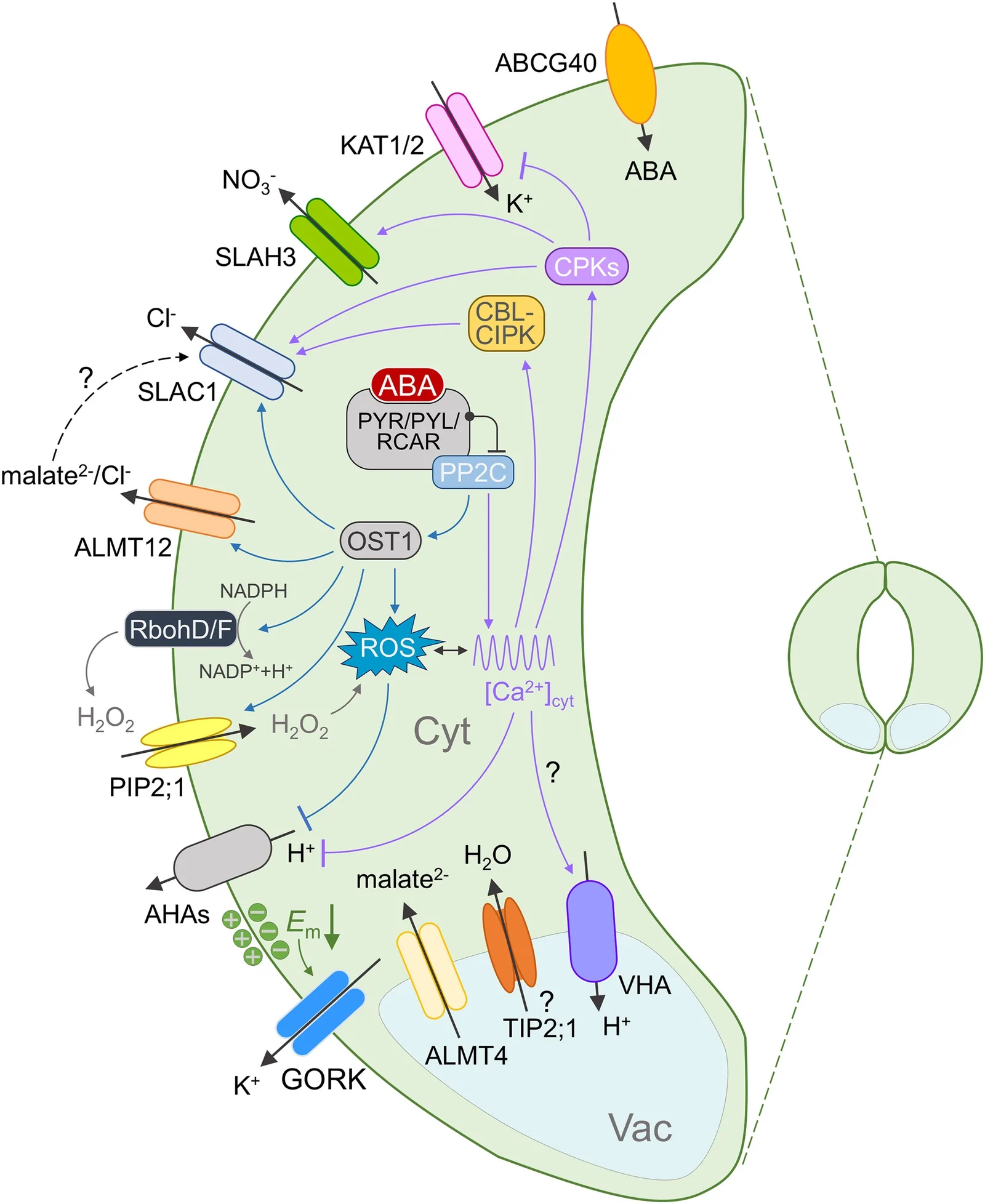
ABA-mediated ion transport and signaling network in guard cells during stomatal closure. In guard cells, ABA can be synthesized locally or taken up by ABCG40 from apoplast (Kang et al. 2010; Merilo et al. 2015). ABA-bound PYR/PYL/RCAR receptors inhibit PP2C/ABI proteins, releasing OST1 through Ca^2+^-independent pathway (blue) and also engaging Ca^2+^-dependent pathways (purple). Active OST1 kinase phosphorylates the S-type anion channel SLAC1 and R-type anion channel ALMT12, respectively promoting chloride (Cl^−^) and malate^2−^ efflux from guard cells. OST1 also enhances RbohD/F-dependent apoplastic ROS/H_2_O_2_ production. H_2_O_2_ is subsequently taken up by OST1-activated PIP2;1 to trigger cytosolic Ca^2+^ signaling. Elevated cytosolic Ca^2+^ ([Ca^2+^]_cyt_) activates CPK- and CBL-CIPK-dependent pathway: CPK23/15 and CBL1-CIPK23 activated SLAC1-mediated Cl^−^ efflux, and CPK23 SLAH3-mediated nitrate (${\mathrm{NO}}_{3}^{-}$) efflux. CPK13 also suppresses K^+^ uptake through KAT1/2. This ABA-dependent ROS and Ca^2+^ signaling can suppress plasma membrane proton (H^+^)-ATPase (AHA) activity by promoting their dephosphorylation and reducing H^+^ extrusion (Kinoshita et al. 1995; Zhang et al. 2004). In addition, vacuolar H ^+^ -ATPase (VHA)-dependent vacuolar acidification also contributes to ABA-induced stomatal closure in guard cells (Bak et al. 2013) and may be coupled with ABA-dependent Ca^2+^ signaling, as indicated by a question mark. Together, anion efflux, inhibited K^+^ uptake, and ROS-suppressed AHA promote plasma membrane depolarization (*E*_m_↓) and GORK-mediated K^+^ efflux. Here, ABA-mediated ROS and Ca^2+^ signaling are interconnected (Kong et al. 2016). The dashed arrow with a question mark indicates an unresolved regulatory interaction in which extracellular malate exported via ALMT12 may modulate SLAC1 activity during ABA signaling (Xu, 2026). Another question mark indicates a possible vacuolar water release by TIP2;1 that requires further investigation (MacRobbie 2006; Tyerman et al. 2021). Cyt, cytosol; Vac, vacuole.

In parallel, OST1 directly phosphorylates and activates SLAC1 (Vahisalu et al 2008; Geiger et al 2009) and ALMT12 (Imes et al 2013) via phosphorylation, promoting guard cell anion efflux. ABA-induced Ca^2+^ signaling further reinforces anion release through Ca^2+^-dependent protein kinases (CPKs) and/or calcineurin B-like protein (CBL) paired with CBL-CIPK (CBL-interacting protein kinase), which phosphorylates and activate SLAC1 and SLAH3 (Mori et al 2006; Maierhofer et al 2014; Shen et al 2025). In addition, CPK, such as CPK13, can inhibit potassium (K^+^) uptake through voltage-gated inward-rectifying K^+^ channels (KAT1) (Ronzier et al 2014), restricting K^+^ accumulation. ABA also activates anion release from vacuoles from ALMT4 (Eisenach et al 2017), reducing osmolyte storage in guard cells. Together, AHA inhibition, anion efflux, suppressed K^+^ uptake, and vacuolar solute release depolarize the *E*_m_ in guard cells. This *E*_m_ depolarization activates guard cell outward-rectifying K^+^ channels (GORK), promoting K^+^ efflux (Hosy et al 2003; Kollist et al 2014) (Fig. 2). The combined loss of K^+^ and anions reduces guard cell osmotic content and turgor pressures, driving water efflux from both the cytosol and vacuole. This vacuolar water release may involve TIP2;1, and its contribution requires further investigation (MacRobbie 2006; Tyerman et al 2021). Although the plasma membrane ion efflux during ABA-mediated stomatal closure is relatively well characterized, this process must be coordinated with ion and water release across the vacuolar membrane. However, this part of mechanisms that integrate these two membrane systems remain elusive (Cubero-Font and De Angeli 2021).

In this process, GORK is primarily activated by membrane depolarization during ABA-induced closure (Kollist et al 2014). Although direct GORK activation by Ca^2+^-dependent CBL1/9-CIPK5 modules was demonstrated in *Xenopus laevis* oocytes, rather than in guard cells, genetic evidence linked this module primarily with jasmonate- and wounding-induced stomatal closure (Förster et al 2019). Additionally, the regulation of SLAC1 appears context dependent: beyond OST1/CPK/CBL-CIPK-mediated SLAC1 activation, extracellular malate transported via ALMT12 may also activate SLAC1; however, whether this ALMT12-drived malate signal is integrated in ABA-mediated guard cell signaling remains unclear (Meyer et al 2010; Mimata et al 2022; Xu, 2026) (Fig. 2).

Beyond controlling ion transporter and channel activity, ABA can alter transport capacity by regulating membrane protein abundance and localization through vesicular trafficking (Jezek and Blatt 2017; Wang et al 2020). In guard cells, ABA facilitates selective endocytosis of KAT1, while the SNARE family protein SYP121 promotes its recycling to the plasma membrane, thereby influencing K^+^ uptake and stomatal opening (Eisenach et al 2012; Yu et al 2026). SNARE-dependent mechanisms also regulate plasma membrane H^+^-ATPases. ABA enhances SYP132-dependent endocytic trafficking of AHA1 from the plasma membrane toward endomembrane compartments (Xia et al 2019). In addition, ABA increases plasma-membrane-associated vesicle-associated membrane protein 711 (VAMP711) and promotes its interaction with AHA1/2, then suppressing H^+^-ATPase activity (Xue et al 2018). As ABA-regulated transporter trafficking has been mostly studied in guard cells and well-reviewed elsewhere (Jezek and Blatt 2017; Wang et al 2020), we only discuss it briefly before turning to ABA-mediated stomatal regulation across multiple cell types.

This guard cell–centered framework is also supported in monocots. Cell-type transcriptome profiling from barley and wheat identified guard cell-specific and guard cell-complex (guard cells plus subsidiary cells) enriched transcripts, reinforcing the core roles of ABA signaling in guard cells for stomatal regulation (Schäfer et al 2018; Wang et al 2021). However, ABA-modulated stomatal control is not solely a guard cell-autonomous process. It is coordinated with broader water and carbon regulation across multiple cell types, including root-to-shoot signaling, bundle sheath cells, and mesophyll cells (Mott et al 2008; Torne-Srivastava et al 2024; da Silva et al 2025). Collectively, we propose that ABA-mediated stomatal responses operate within a multicellular signaling network in plants.

## ABA coordinates stomatal regulation through root-to-shoot and bundle sheath signal transduction

Roots perceive soil water status and adjust shoot transpiration through long-distance root-to-shoot signaling (Scharwies and Dinneny 2019). Under drought, sulfate (${\mathrm{SO}}_{4}^{2-}$) acts as an early root-to-shoot chemical signal transported through the xylem, promoting guard cell–autonomous ABA biosynthesis and stomatal closure (Ernst et al 2010; Malcheska et al 2017). In guard cells, ${\mathrm{SO}}_{4}^{2-}$ induces *NCED3* expression and is assimilated into cysteine through the plastid cysteine synthase complex; then, cysteine supports MCSU/ABA3-dependent AAO3 activation and local ABA biosynthesis (Malcheska et al 2017; Batool et al 2018; Sun et al 2025, 2026). In addition, root-derived *CLAVATA3/ESR-RELATED 25* (*CLE25*) peptides move through the vascular tissue from roots to shoots, where they are perceived by receptor-like kinases BARELY ANY MERISTEM 1 (BAM1) and BAM3 to promote *NCED3* expression, stimulating ABA biosynthesis, stomatal closure, and enhanced water use efficiency in *Arabidopsis* (Takahashi et al 2018). By contrast, locally produced CLE5 dodecapeptide (CLE5p) can promote drought-induced stomatal closure without inducing ABA biosynthesis (Shimotohno et al 2025).

Within this *CLE25-NCED3* pathway, vascular *NCED* expression provides a useful transcriptional reporter of leaf ABA biosynthesis (Xiong and Zhu 2003; Takahashi et al 2018), consistent with the evidence that drought-induced ABA biosynthesis occurs mainly in vascular parenchyma cells and other vein-associated tissues, rather than uniformly across the leaf (Endo et al 2008). ABA can also move from root to shoot to regulate stomatal aperture via ATP-binding cassette (ABC) transporter ABCG25, but its role in drought-induced stomatal closure is likely limited in *Arabidopsis* (Yang et al 2024). In some circumstances, local ABA biosynthesis can reduce dependence on long-distance ABA transport. For instance, soil-drying closing stomata in tomato (*Solanum lycopersicum*) cv. *Rheinlands Ruhm* does not require root ABA production (Holbrook et al 2002), while drought-induced ABA accumulation in soybean (*Glycine max*) roots relies more on local NCED-dependent ABA synthesis, rather than on shoot-to-root ABA import (Puértolas et al 2026). Together, these findings indicate that stress-induced ABA accumulation can involve local biosynthesis, long-distance signaling, and ABA transport.

At the leaf scale, ABA coordinates both water transport and water loss by regulating leaf hydraulic conductance (*K*_leaf_) and stomatal conductance (*g*_s_). *K*_leaf_ reflects water flow through the leaf from the xylem toward the evaporative sites (e.g. stomata), whereas *g*_s_ determines water vapor diffusion from leaf airspaces to the atmosphere through stomatal pores. As such, *K*_leaf_ and *g*_s_ are tightly coupled, linking xylem water supply with transpirational water loss via stomatal pores in response to environment (e.g. blue light) and endogenous signals (e.g. ABA) (Chaumont and Tyerman 2014; Buckley 2019; Scharwies and Dinneny 2019). In this process, blue light is perceived by phototropins, which activate AHA in both bundle sheath cells and guard cells. In bundle sheath cells, activation of AHA2 drives H^+^ extrusion, increases cytosolic pH (pH_cyt_), and acidifies the surrounding apoplast/xylem sap; this is associated with enhanced *K*_leaf_ thereby facilitating radial water movement from the xylem toward the leaf airspace (Grunwald et al 2021, 2022). Concurrently, blue-light activation of AHA1 and AHA2 promote stomatal opening and increases *g*_s_ (Kinoshita et al 2001; Fuji et al 2024) (Fig. 3). By contrast, ABA inhibits AHA2-depedent acidification of the apoplast, increases apoplastic pH (pH_apo_), and promotes vacuolar H^+^ sequestration by VHA, such as VHA-a2 and VHA-a3, and consequently the reduced *K*_leaf_ (Torne-Srivastava et al 2024), which may partially involve pH-dependent regulation of aquaporins (Tyerman et al 2021). In guard cells, ABA similarly inhibits AHA1/2, promotes vacuolar H^+^ pumping, contributing to cytosolic alkalinization, stomatal closure and reduced *g*_s_ (Yamauchi et al 2016; Pei et al 2022; Torne-Srivastava et al 2024). Consistent with this model, impairment of ABA signaling in bundle sheath cells results in greater *K*_leaf_ and *g*_s_ (Yaaran et al 2023), underscoring that ABA-mediated stomatal regulation is not restricted to guard cells but also depends on hydraulic and signaling inputs from surrounding leaf tissues (Fig. 3).

**Figure 3:**
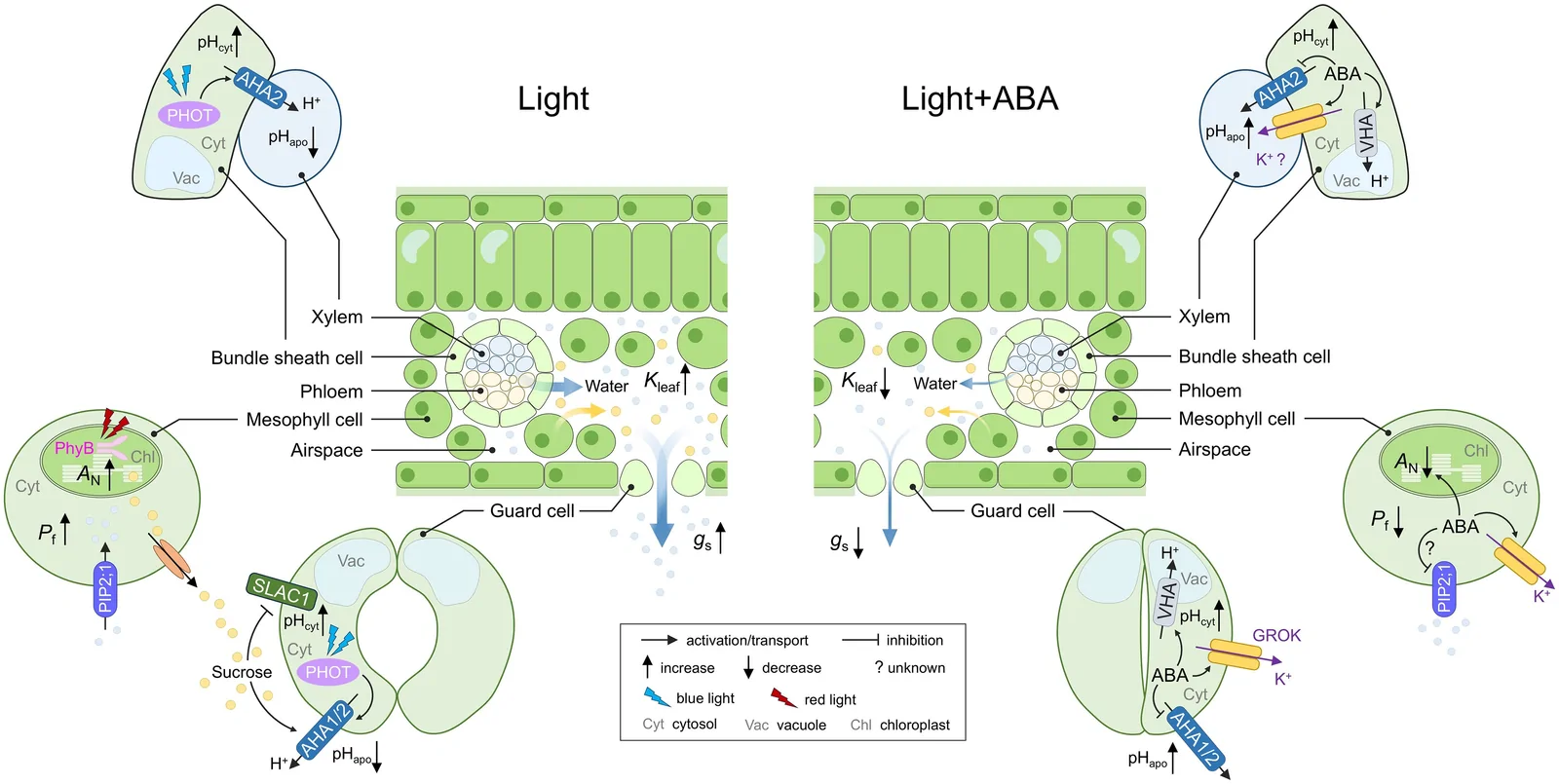
Proposed multicellular coordination of light- and ABA-mediated stomatal movement, water transport and gas exchange. (Left) Under light conditions, blue light perceived by phototropins (PHOTs) activates AHAs in bundle sheath cells (e.g. AHA2) and guard cells (e.g. AHA1/2). In bundle sheath cells, AHA2-mediated H^+^ extrusion acidifies the apoplast (pH_apo_ ↓) and increases cytosolic pH (pH_cyt_ ↑), which is associated with enhancing leaf hydraulic conductance (*K*_leaf_) and water delivery from the xylem toward evaporative sites adjacent to the leaf airspace. In guard cells, AHA1/2 activation by light promote stomatal opening, increasing stomatal conductance (*g*_s_) and water vapor diffusion from leaf airspace to atmosphere (Kinoshita et al. 2001; Yamauchi et al. 2016; Grunwald et al. 2022; Torne-Srivastava et al. 2024). Light also maintains high osmotic water permeability (*P*_f_) in mesophyll cells partially through plasma membrane PIP2;1-mediated water transport (Prado et al. 2013; Negin et al. 2019; Tyerman et al. 2021; Yaaran et al. 2023). In parallel, red-light/phytochrome B (PhyB) signaling may enhance mesophyll CO_2_ assimilation (A_N_) and sucrose production. Mesophyll-derived sucrose may reach the apoplast through an unresolved export pathway, in which extracellular sucrose has been reported to promote H ^+^ -ATPase (e.g. AHA1) phosphorylation and H^+^ pumping in guard cells, while inhibiting SLAC1-mediated anion currents, thereby supporting stomatal opening (Flütsch and Santelia 2021; Zait et al. 2025). (Right) In the presence of ABA, light-induced water flow and stomatal opening are counteracted through coordinated effects in guard cells, bundle sheath cells, and mesophyll cells. ABA inhibits plasma membrane AHA activity and stimulates tonoplast VHA-mediated H^+^ sequestration in bundle sheath cells and guard cells, promoting both cytosolic and apoplastic alkalinization (pH_cyt_ ↑ and pH_apo_ ↑) and reducing *K*_leaf_ and *g*_s_. ABA also triggers K^+^ efflux through GORK in guard cells and promoting stomatal closure. In mesophyll cells, ABA reduces *P*_f_, possibly by inhibiting PIP2;1-dependent water transport that remains to be fully confirmed, and ABA also reduces photosynthetic A_N_ (Prado et al. 2013; Grondin et al. 2015; Tyerman et al. 2021), which may potentially reduce sucrose-mediated promotion of stomatal opening (Zait et al. 2025). ABA-induced K^+^ efflux from mesophyll cells has been reported, although the responsible channel is yet unidentified (Mak et al. 2014). Whether ABA similarly regulates K^+^ efflux across bundle sheath cells remains unresolved (Torne-Srivastava et al. 2024) Question marks indicate unresolved mechanisms or transport components. Chl, chloroplasts; Cyt, cytosol; Vac, vacuole.

Additionally, it was shown that neither low (1 mM) nor high (11 mM) K^+^ supplement through the leaf petiole altered xylem sap pH in *Arabidopsis*. However, alkalinizing xylem sap by vanadate required high K^+^ supplementation (Grunwald et al 2021), suggesting that optimal xylem K^+^ homeostasis may facilitate xylem sap alkalinization mediated by AHA inactivation. Other studies found that bundle sheath cells maintained a more hyperpolarized *E*_m_ (Wigoda et al 2017) and accumulated higher K^+^, compared with other cell types, such as mesophyll cells (Conn and Gilliham 2010), a process potentially linked to AHA2 activity in bundle sheath cells (Wigoda et al 2017). Given that ABA stimulates K^+^ efflux to the guard cell apoplast during stomatal closure (Blatt 2024), it remains unclear whether ABA-induced AHA2 inactivation and membrane depolarization also alters K^+^ flux across bundle sheath cells to the xylem, potentially facilitating xylem sap alkalinization (Fig. 3).

## Mesophyll ABA signaling links stomatal regulation with photosynthesis and metabolism in leaves

An early study demonstrated that guard cell protoplasts maintain higher ABA concentrations than mesophyll protoplasts in *Vicia faba* (Lahr and Raschke 1988). In *Arabidopsis*, vascular tissues and guard cells are established sites for ABA biosynthesis, whereas the extent to which mesophyll cells contribute to ABA biosynthesis remains less clear (Bauer et al 2013; Merilo et al 2015; Yaaran et al 2019). However, ABA signaling in mesophyll cells also contributes to stomatal regulation (Negin et al 2019; Yaaran et al 2023). Negin et al (2019) used a mesophyll-specific promoter to drive expression of a dominant mutant *ABI1* allele (*abi1-1*) in *Arabidopsis*, generating *FBPase::abi1-1*/MCabi (Yaaran et al 2023). In MCabi plants, foliar ABA application failed to strongly induce *NCED3*, confirming impaired ABA signaling in mesophyll cells (Negin et al 2019). Under control conditions, these lines displayed >25% higher *g*_s_ and ∼10% greater photosynthesis (CO_2_ assimilation, A_N_) than wildtype plants, despite similar Rubisco carboxylation capacity (Negin et al 2019; Yaaran et al 2023) and also showed higher *K*_leaf_ (Yaaran et al 2023). Although ABA reduced *K*_leaf_, *g*_s_ and photosynthesis in both genotypes, MCabi consistently maintained higher values under ABA treatment (Negin et al 2019; Yaaran et al 2023) (Fig. 3). Similarly, two plasma membrane ABC transporters, ABCG17 and ABCG18, have been associated with ABA import into *Arabidopsis* mesophyll cells. Artificial microRNA knockdown of both genes reduced ABA uptake into mesophyll cells of *mir17,18* lines, increasing ABA accumulation in guard cells and reducing stomatal pore opening under nonstress conditions. Under drought, both *ABCG17* and *ABCG18* were downregulated, potentially reducing ABA import into mesophyll cells and promoting guard cell ABA accumulation and stomatal closure (Zhang et al 2021). Taken together, these studies highlight an important role for ABA accumulation and sensing in mesophyll cells in controlling stomatal behavior.

Mesophyll photosynthetic metabolism also influences stomatal behavior. In intact leaves, stomata of *Tradescantia pallida* and *Pisum sativum* responded rapidly to short-term changes in environmental light and CO_2_; however, these responses were significantly reduced in isolated epidermal peels and recovered when the epidermis was placed in contact with mesophyll layers. This provided early evidence that mesophyll-derived signals contribute to stomatal regulation to light and CO_2_ (Mott et al 2008). Consistently, in *Commelina communis*, stomata in leaf segment opened rapidly under red light; however, this opening response became much weaker in isolated epidermal strips and was reinstated when the epidermis was placed on mesophyll tissue. Furthermore, inhibition of photosynthesis by DCMU greatly suppressed red light–induced stomatal opening. Together, these findings support a stronger contribution of mesophyll photosynthesis to stomatal opening under red light (Fujita et al 2013). A multispecies meta-analysis further linked photosynthesis-derived sugars—such as sucrose, glucose, and fructose—to stomatal regulation (Zait et al 2025). A recent report showed that red light–driven mesophyll photosynthesis increases apoplastic sugars in *Arabidopsis* and *V. faba* with sucrose reaching 1 to 2 mM concentrations (Zait et al 2025). Extracellular sucrose promoted AHA1 phosphorylation and inhibited SLAC1-mediated anion currents in guard cells, thereby proposed to facilitate stomatal opening under red light (Flütsch and Santelia 2021; Zait et al 2025) (Fig. 3). However, the pathway by which photosynthesis-derived sugars are exported from mesophyll cells into the apoplast remains unresolved (Zait et al 2025) (Fig. 3), and it is not clear yet whether and how ABA modulates apoplastic sucrose availability/signaling (e.g. MCabi line). Because ABA influences phloem sugar loading and unloading (Gong et al 2015; Braun 2022), it may also alter leaf apoplastic sucrose availability. Thereby, we present the possible link that impaired ABA sensing in MCabi plants may increase photosynthetic carbon assimilation and potentially apoplastic sucrose. Such increased sucrose availability could in turn promote AHA1-associated H^+^ extrusion and suppress SLAC1-mediated anion efflux in guard cells, thereby contributing to greater stomatal opening and higher *g*_s_ as observed in MCabi plants (Negin et al 2019; Yaaran et al 2023; Zait et al 2025) (Fig. 3). This proposed mesophyll-ABA linked guard cell–sucrose pathway remains to be tested.

Intriguingly, this multicellular complexity of ABA signaling extends even further: responses are also heterogeneous within nominally uniform cell types. For instance, *Arabidopsis* spongy mesophyll protoplasts displayed a higher membrane osmotic water permeability (*P*_f_) than palisade mesophyll protoplasts (∼20 µm s^−1^ in spongy mesophyll protoplasts vs. ∼13 µm s^−1^ in palisade mesophyll protoplasts). ABA reduced the *P*_f_ of spongy mesophyll protoplasts by up to 60%, whereas the *P*_f_ of palisade mesophyll protoplasts was largely unaffected even at high ABA concentrations up to 50 µM (Yaaran et al 2023). Yaaran et al (2023), therefore, proposed that higher basal *P*_f_ in spongy mesophyll cells may allow them to more directly regulate water movement from the apoplast toward the sites of evaporation during transpiration; while their greater ABA sensitivity may enable more rapid restriction of leaf water flux during stress (Fig. 3). Similarly, abaxial (lower surface) stomata are more responsive to light than adaxial (upper surface) stomata in *Arabidopsis*, linked with differential abundance of K^+^ channels on each surface (Wei et al 2025). Such comparable surface-dependent differences in stomatal behaviors have been also reported in other species, including a greater ozone (O_3_)-induced reduction in *g*_s_ on the adaxial surface in 3 poplar (*Populus deltoides* × *P. nigra*) genotypes and stronger ABA response of abaxial stomata in *C. communis* and *V. faba* (Pemadasa 1981; Wang et al 1998). Taken together, we propose that stomatal regulation is coordinated by multicellular ABA signaling across guard cells, bundle sheath cells and mesophyll cells, which can be better understood in a surface-specific context as part of cellular heterogeneity.

## Single-cell and spatial omics advance knowledge in multicellular coordination of ABA signaling

Cell type–specific transcriptomic approaches (e.g. guard cell protoplasts [Leonhardt et al 2004; Pandey et al 2010; Zhao et al 2019; Wei et al 2025] and intact guard cells [Bates et al 2012]) have been instrumental in dissecting ABA-mediated signaling pathways in guard cells underlying stomatal regulation and gas exchange. Recently, Wei et al (2025) demonstrated that the distinct light responsiveness in Arabidopsis guard cells of adaxial and abaxial stomata arises, at least in part, from differences in relative abundance of two inward rectifying K^+^ channels: KAT1 and AKT1. Single-cell RNA sequencing (scRNA-seq) of guard cell protoplasts isolated separately from the two leaf surfaces showed that both genes are expressed in guard cells, but *KAT1* is enriched by >2-fold in abaxial guard cells, whereas *AKT1* is more abundant on the adaxial guard cells. However, these processes are systemically coordinated across multiple leaf cell types, rather than by guard cells alone. Consequently, transcriptomic analyses that focused on a single isolated cell type cannot readily resolve multicellular organization, cell-cell interactions, or the spatial separation between ABA biosynthesis and ABA-responsive elements within intact tissue.

Recent advances in scRNA-seq, especially when integrated with spatial transcriptomics approaches, now allow reconstruction of plant cell atlases and provide a framework for examining how hormone-regulated responses are distributed across different cell types simultaneously (Yu et al 2023; Adema et al 2024; Tao et al 2026). A compelling ABA-relevant example is provided by a recent rice root study of soil compaction (Zhu et al 2025), a condition in which soil becomes more densely packed and mechanically resistant (Pandey and Bennett 2024). In this study, a reference rice root atlas of >79,000 scRNA-seq profiles was generated to compare transcriptomic responses between compacted and noncompacted soil conditions, revealing that ABA biosynthesis genes (e.g. *OsAAO1*, *OsNCEDs*, *OsZEP/ABA1*) were upregulated predominantly in phloem-associated inner vascular tissues; whereas ABA-responsive genes, including *pathogenesis-related genes* (*OsPR10s*), *dehydrin Rab16C* (*OsRAB16C*), and *abscisic stress-ripening protein 5* (*OsASR5*), were enriched mainly in outer root layers, especially exodermis. In parallel, compacted soil also induced cell wall–related genes in exodermis, including xylanase-inhibitor genes, *xyloglucan endotransglucosylase 22* (*OsXTH22*) and *xylan 3-O-Arabinosyltransferase 7* (*OsXAT7*). Functional analysis of ABA-biosynthetic rice mutants further showed that ABA biosynthesis is induced in inner root layers and required for compaction-induced lignin and suberin deposition, barrier formation, and reduced radial water loss in outer root layers (Zhu et al 2025). Thus, it revealed that that scRNA-seq, spatial validation, and genetics together separated the cellular domain of ABA production from the domains of ABA-responsive transcription and downstream adaptive barrier formation (Wang and Dinneny 2025).

Notably, this study also illustrates how spatial omics methods can complement scRNA-seq analysis. In the initial scRNA-seq analysis, transcriptomic profiles were clustered according to transcriptomic similarity, and provisional cell identities were assigned using previously validated marker genes (Zhu et al 2025). Because many clusters still lacked confident annotation, Zhu et al (2025) validated more than 40 cell-type markers using multiplexed fluorescence in situ hybridization to refine the cell-type assignments. More broadly, this example aligns with growing evidence that spatial information can guide interpretation of scRNA-seq datasets, particularly because unsupervised scRNA-seq pipelines often focus on dominant sources of variation and may fail to resolve spatially distinct cell types when differential transcriptional profiles are limited (Yu et al 2023; Adema et al 2024).

The same issue is likely relevant to leaf dorsiventrality and stomatal regulation (Driesen et al 2023; Hõrak 2026). *Arabidopsis* stomatal regulation is likely coordinated unequally across the two leaf surfaces, with spongy mesophyll cells and abaxial guard cells contributing differently from palisade mesophyll cells and adaxial guard cells, then dissociation-based scRNA-seq alone may be insufficient to resolve the relevant mesophyll or epidermal cell types (Adema et al 2024). Tenorio Berrío et al (2022) generated an early single-cell atlas of young developing *Arabidopsis* leaves and 14 differential cell populations; however, within the mesophyll cluster, spongy mesophyll-associated marker gene, trans*-acting siRNA 3* (*TAS3*) (Chitwood et al 2009), and palisade mesophyll-associated marker gene, *axial regulator YABBY 1* (*YAB1*) (Uemoto et al 2018), did not define clearly separated unsupervised clusters. Likewise, In mature *Arabidopsis* leaves, Xia et al (2022) showed that scRNA-seq alone could not distinguished upper versus lower epidermal cells or spongy versus palisade mesophyll cells, but integration of spatial transcriptomes resolved these subtypes. Together, these studies suggest that resolving multicellular ABA coordination in leaves will probably leverage not only scRNA-seq, but also spatially resolved or spatially supervised single-cell approaches (Fig. 4).

**Figure 4:**
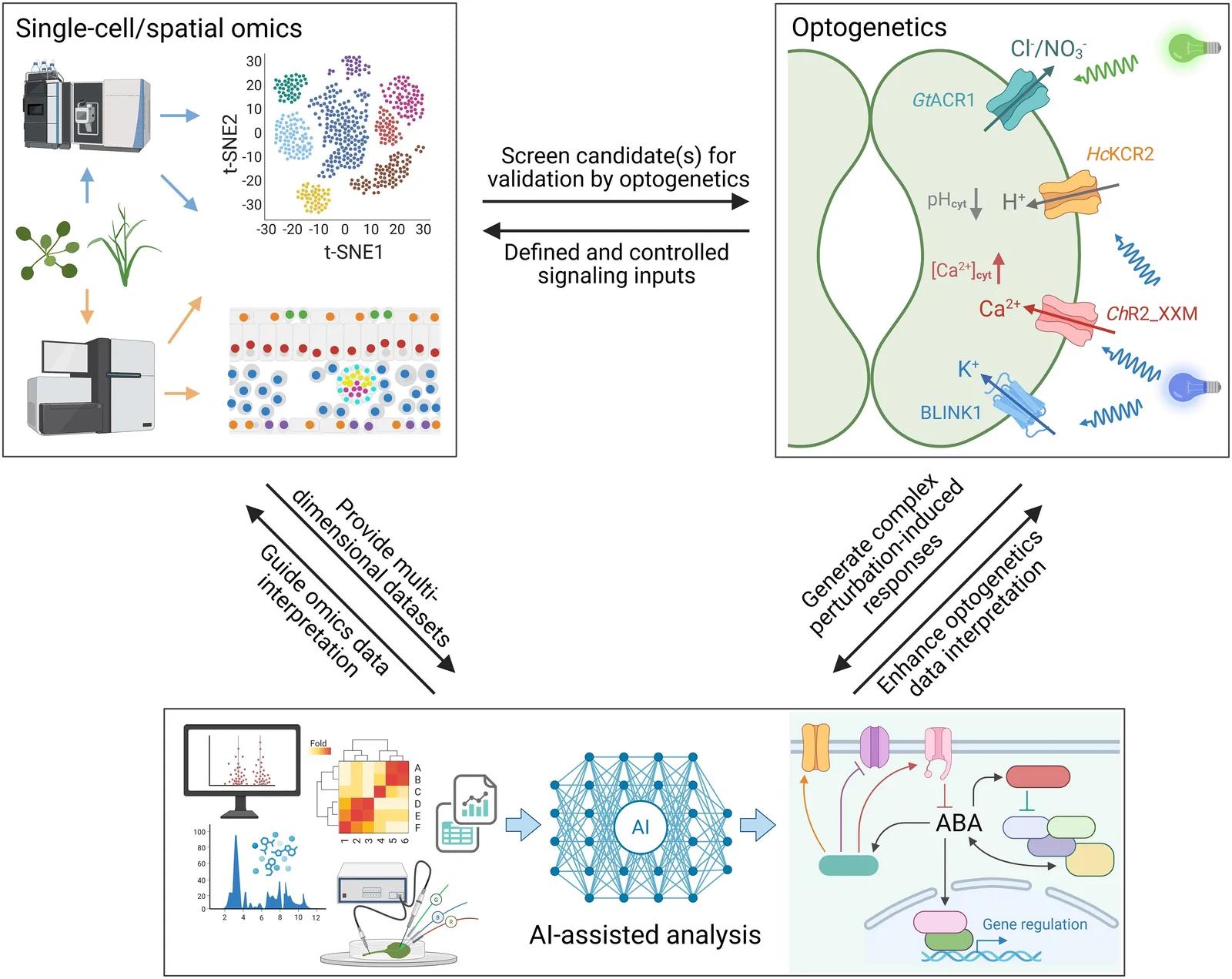
Proposed perspective of combing single-cell/spatial omics, optogenetics, and AI-assisted analysis to dissect multicellular ABA signaling. Single-cell and spatial omics can identify cell-specific ABA-mediated genes, metabolites, candidate transporting processes and signaling modules (Xia et al. 2022; Adema et al. 2024; Zhu et al. 2025); these candidates can be functionally characterized and tested using optogenetics tools that impose defined ion flux and signaling inputs in guard cells by different light conditions, such as *Gt*ACR1-mediated Cl^−^/${\mathrm{NO}}_{3}^{-}$ efflux (Huang et al. 2021), *Hc*KCR2-mediated H^+^ influx and cytosolic pH (pH_cyt_) regulation (Huang et al. 2024), *Ch*R2-XXM-mediated Ca^2+^ influx and elevated cytosolic Ca^2+^ ([Ca^2+^]_cyt_) concentrations (Huang et al. 2024), or BLINK1-modulated K^+^ transport (Papanatsiou et al. 2019). Conversely, optogenetics can also be used to generate perturbation and defined signaling states, subsequently profiled by single-cell and spatial omics (Hedrich and Gilliham 2025). Both advanced approaches generate multidimensional datasets and complex perturbed responses, which can be interpreted by AI-assisted analysis to empower the dissection of ABA-mediated signaling pathways at single-cell resolution (Kim et al. 2021; Coatsworth et al. 2024). Created in BioRender (https://BioRender.com/waivuca).

Beyond simply cataloging cell-type transcriptomics, integrated spatial transcriptomics and spatial metabolomics could help resolve whether cellular domains of ABA biosynthesis, accumulation, perception, and downstream responses are spatially coincident or separated across leaf cell types (Yu et al 2023; Tao et al 2026). In the context of stomatal regulation, combining these spatial omics could reveal how these cell type–specific processes are coordinated to regulate stomatal behavior, gas exchange, and water movement. However, spatial metabolomics alone is unlikely to directly resolve ion flux, and candidate ABA-regulated transport modules identified through spatial omics will still require physiological and functional validation. Therefore, plant optogenetics provides a complementary toolkit for dissecting such processes, as discussed below (Fig. 4).

## Integrated optogenetics to reveal complex ABA signaling in plants

ABA-mediated ion transport is a central downstream effector for guard cell osmotic adjustment and volume change for stomatal movement, representing best-defined outputs of ABA perception and signaling in guard cells (see reviews by Blatt [2024] and Pantoja [2021] and references therein). These processes have been extensively dissected by electrophysiology since the early 1980s (Pantoja 2021; Blatt 2024), when microelectrodes were first applied to *V. faba* to examine the effect of ABA on *E*_m_, showing that ABA depolarizes the *E*_m_ and providing early evidence that ABA modulates transmembrane ion fluxes in guard cells (Ishikawa et al 1983). More recently, electrophysiology combined with plant optogenetics has further expanded our capability to interrogate ion fluxes and guard cell signaling with causal precision (Konrad et al 2023; Hedrich and Gilliham 2025).

Optogenetics uses genetically encoded photoreceptors to manipulate and define biological processes (Emiliani et al 2022). In plants, these tools include not only microbial rhodopsins, such as channelrhodopsins (CHRs), but also other engineered light-activated ion channels. Higher plants possess complex ion transport network but lack natural light-gated ion channels. These light-responsive ion channels can be exploited to selectively manipulate specific ion fluxes, examine downstream signaling consequences and define physiological outputs (Konrad et al 2023; Hedrich and Gilliham 2025). For instance, guard cell-specific manipulation of synthetic chimeric blue light-induced K^+^ channel (BLINK1) accelerated stomatal kinetics, improved water use efficiency, and enhanced *Arabidopsis* growth (Papanatsiou et al 2019). This strategy is highly relevant to ABA-triggered ion flux and signaling, because ABA, like many other signals, such as ROS (Wu et al 2020), CO_2_ (Webb et al 1996), and pathogen-associated molecular patterns (Thor and Peiter 2014), can trigger increase in cytosolic Ca^2+^ ([Ca^2+^]_cyt_) in guard cells and promote stomatal closure (McAinsh et al 1990; Huang et al 2019). How guard cells decode different Ca^2+^ signatures remained elusive until Huang et al (2024) used a *Chlamydomonas reinhardtii* Ca^2+^-permeable CHR-2 variant (*Ch*R2^D156H^/*Ch*R2-XXM) to impose defined [Ca^2+^]_cyt_ signatures in *N. tabacum* guard cells. A single 0.1-s blue-light pulse triggered a unitary [Ca^2+^]_cyt_ spike and amplified endoplasmic reticulum Ca^2+^ release and activation of S-type anion channels to promote stomatal closure. The stomatal closure scaled with [Ca^2+^]_cyt_ transient number (∼10% stomatal closure after 1 pulse to nearly complete closure after 12 pulses), while higher pulse frequency accelerated stomatal closing speed (Huang et al 2024). Minimal effect on control plants suggests that *Ch*R2-XXM can selectively manipulate Ca^2+^-dependent stomatal responses with limited interference from endogenous light signaling (Huang et al 2024; Hedrich and Gilliham 2025).

The broader use of *Ch*R2-XXM is illustrated by recent applications in *Arabidopsis* roots. Kulich et al (2026) employed the *Ch*R2-XXM to repeatedly execute [Ca^2+^]_cyt_ oscillations, showing that the *Ch*R2-XXM-mediated Ca^2+^ signaling was sufficient to trigger apoplastic ROS bursts, linked to auxin-associated root responses and mechanical navigation during soil growth (Kulich et al 2026). Consistently, Song et al (2025) used it to define [Ca^2+^]_cyt_ inputs that modulated auxin-induced *E*_m_ depolarization, transcriptional responses, and root growth. Similar approaches may be employed to ABA signaling by imposing defined [Ca^2+^]_cyt_ inputs in ABA-signaling mutants to test whether Ca^2+^ can bypass specific signaling defects and separate components acting upstream or downstream of Ca^2+^ signals. This could help determine how ABA-dependent Ca^2+^ signals are perceived into ion transport and stomatal responses across different cell types.

Moreover, other types of light-regulated ion channels have also been adapted in plants. These include anion CHR *Gt*ACR1 from *Guillardia theta*, which is permeable to chloride and nitrate (Cl^−^/${\mathrm{NO}}_{3}^{-}$) (Huang et al 2021), and kalium CHR 2 (*Hc*KCR2) from *Hyphochytrium catenoides*, which drives H^+^ import and alters pH_cyt_ (Huang et al 2023). ABA-mediated stomatal regulation involves a complex, interconnected ion transport network, including K^+^, Cl^−^, ${\mathrm{NO}}_{3}^{-}$, malate^2−^, H^+^, and Ca^2+^ (Pantoja 2021; Blatt 2024) (Fig. 2). Therefore, optogenetics provides a modular toolkit to interrogate major ABA-relevant ion fluxes without activating the entire ABA pathway at once (Hedrich and Gilliham 2025). In principle, such tools could be optimized to isolate individual ABA-regulated ion transport processes through bypassing upstream ABA perception or counteracting specific ABA-triggered events without disrupting other branches. For example, ABA is known to induce fast cytosolic alkalization in guard cells, followed by an increase in [Ca^2+^]_cyt_ to close stomata (Irving et al 1992; Li et al 2021). In this scenario, *Hc*KCR2 may be used as a light-gated tool to acidify pH_cyt_ (Huang et al 2023), possibly attenuating ABA-induced cytosolic alkalinization in guard cells and enabling to test which types of ABA-regulated ion-transport events require this pH_cyt_ shift (Fig. 4). Similar strategies could be also extended to test ABA-induced pH_cyt_ shift in mesophyll cells and bundle sheath cells to determine whether pH_cyt_-dependent K^+^ efflux, anion transport, or [Ca^2+^]_cyt_ transients operate upstream, downstream, or in parallel with ABA-triggered pH_cyt_ regulation in comparison with guard cells.

Finally, ABA signaling is often interconnected with other signal elements, including pH, nitric oxide, ROS, and Ca^2+^, to shape physiological responses (Pei et al 2000, 2022; Wang et al 2013; Chen et al 2016; Torne-Srivastava et al 2024). Multiple CHRs that respond to different light wavelengths (e.g. *Gt*ACR1 and *Ch*R2-XXM) (Ding et al 2024) can be combined and activated sequentially or simultaneously to separate and manipulate ABA-associated signaling modules, defining their individual and synergistic effect (Hedrich and Gilliham 2025).

In summary, spatial/single-cell omics and optogenetics could be complementary approaches to advance ABA research in different cell types in plants. Spatial and single-cell omics can identify ABA-regulated candidates, metabolites, and transport modules across guard cells, bundle sheath cells, and mesophyll cells, and optogenetics can test their physiological contribution by selectively manipulating defined ion fluxes or signaling events. Also, optogenetic manipulation of ion fluxes may generate controlled or defined signaling states that are then profiled by spatial transcriptomics, spatial metabolomics, or scRNA-seq, enabling the discovery of novel cell type–specific ABA-mediated response and intracellular signaling pathways.

## Systems modeling and AI-assisted approaches to decipher ABA signaling in plants

Guard cells are among the most studied plant cell types and have also been extensively investigated with several mathematical modeling approaches to address complex guard cell signaling networks (Albert et al 2017), stomatal CO_2_ sensing mechanisms (Hashimoto et al 2006; Hu et al 2010; Xue et al 2011; Hsu et al 2018; Gan et al 2024), and ion transport dynamics (Chen et al 2012; Hills et al 2012; Huang et al 2021; Jezek et al 2021). For instance, by combining computational modeling of OnGuard3 platform with genetic perturbation, Wei et al (2025) predicted that KAT1 supports stomatal opening effectively, helping to explain differential light responses between adaxial and abaxial stomata. Similarly, Huang et al (2021) used GuardIon model to interpretate how GtACR1-mediated anion efflux and *E*_m_-depolarization activated GORK-like K^+^ efflux, interacts with H^+^-ATPase-mediated H^+^ pumping, and ultimately changes osmotic turgor in guard cells to close stomata in *N. tabacum*. However, these models mostly focus on guard cells (Huang et al 2021; Jezek et al 2021; Wei et al 2025), highlighting the need to extend modeling frameworks to ion transport and signal in/outputs across interacting cell types, such as mesophyll cells and bundle sheath cells.

AI-assisted approaches, such as AlphaFold 3 modeling (Abramson et al 2024), are beginning to turbo charge ABA-signaling research. ABA-mediated stomatal closure is not restricted to seed plants, but also occurs across diverse fern species, including *Ceratopteris richardii* (Plackett et al 2021), *Polystichum proliferum*, *Nephrolepis exaltata* (Cai et al 2017), *Athyrium filix-femina*, and *Dryopteris carthusiana* (Hõrak et al 2017). Analysis of ABA signaling homologues suggests ABA-responsive stomatal regulation occurs at least in some early vascular land plants (Cai et al 2017; Hõrak et al 2017). Early structural and biochemical analysis established that *Arabidopsis* PYR/PYL/RCAR receptors can function in dimeric or monomeric forms with distinct ABA sensitives, with monomeric receptors showing higher ABA-binding affinity than dimeric forms. Mutagenesis, generating PYR1^H60P^, further identified H^60^ as a key residue determine receptor's oligomeric state (Dupeux et al 2011). This principle was later extended across plant species using AI-assisted structural analysis. More recently, Rivera-Moreno et al (2026) investigated the structural evolution of the PYR/PYL/RCAR ABA receptors, including CsPYL1 and MpPYL1 respectively from an angiosperm (*Citrus sinensis*) and a liverwort (*Marchantia polymorpha*). CsPYL1 functions as an ABA-dependent dimeric receptor with lower ABA sensitivity (half-maximal inhibitory concentration, half-maximal inhibitory concentration = 230 nM), which is proposed to contribute to signaling at higher ABA levels during acute drought. In contrast, MpPYL1 is monomeric with high ABA sensitivity (half-maximal inhibitory concentration = ∼70 nM), and proposed to sense and decode mild stress-induced ABA concentration and signaling (Dupeux et al 2011; Rivera-Moreno et al 2026). AlphaFold-based prediction identified a 5-residue signature that contributes to the receptors’ oligomeric state and ABA sensitivity. Reciprocal amino acid residue substitutions generated CsPYL1Mp (CsPYL1^I91L/V112L/V192L/L195C/K199S^) and MpPYL1Cs (MpPYL1^L91I/L111V/L191V/C194L/S198K^), respectively converting CsPYL1Mp toward MpPYL1-like monomeric, high ABA sensitivity and MpPYL1Cs toward CsPYL1-like dimeric, low affinity (Rivera-Moreno et al 2026). This provides an example of AI-assisted ABA receptors engineering, although whether such engineered receptors improve plant stress adaptation remains to be demonstrated.

Moreover, spatial and single-cell omics are expected to generate large, high-dimensional datasets that capture cell type–specific transcriptional, metabolic, and spatial responses, AI-assisted analysis can support the interpretation of these datasets by resolving cellular heterogeneity, identifying stress- and hormone-mediated modules, and prioritizing candidate genes, metabolites, and cell states associated with signaling outputs (Alexandrov 2023; Sanchez-Munoz et al 2025; Tao et al 2026). For example, Sanchez-Munoz et al (2025) integrated meta-analysis and unsupervised machine learning algorithm to analyze multitranscriptomics in *Arabidopsis* across multiple stress, tissue, and time points. This study identified a core set of stress-related gene families involved in ethylene response, such as *Expansins* (*EXP*), *Ethylene response factor* (*ERF*), *WRKY*, *Mitogen-activated protein kinase* (*MAPK*), supporting ethylene as a central regulatory component of broad abiotic-stress transcriptional responses (Sanchez-Munoz et al 2025). Similar strategies could be adapted to analyze ABA-mediated signaling at single-cell resolution (Fig. 4).

AI-assisted analysis may also complement optogenetics by converting complex perturbation-induced responses into quantitative, interpretable features, as demonstrated previously in mammals (Kim et al 2021; Hyeon et al 2025). For instance, a recent study by Hyeon et al (2025) combined optogenetics and AI-driven analysis to provide an efficiency diagnose of extensive behavior evaluation of mice (*Mus musculus*) with Parkinson's disease. Although direct integrative studies of optogenetics and AI remains limited in plants, Coatsworth et al (2024) provides a relevant precedent by using time-resolved root electrochemical sensing data to train a machine learning model that predicts ion-uptake behavior. This approach could be extended to ion transporter mutants to predict how altered transport mechanisms affect the overall ion uptake (e.g. ${\mathrm{NO}}_{3}^{-}$) (Coatsworth et al 2024), and demonstrates how AI can extract ion transport features from complex physiological outputs to guide the discovery of new ion transport regulatory mechanisms.

Taken together, AI-assisted approaches can accelerate ABA research by linking structural modeling, spatial/single-cell omics and optogenetics perturbation to identify, prioritize, and interpret ABA-responsive modules across multiple cell types and convert complex signaling and physiological outputs into multicellular ABA regulations (Fig. 4).

## Concluding remarks and future perspectives

Over 6 decades after ABA was first discovered, major progress has clarified how this phytohormone regulates development, growth, stress responses and environmental adaptation. Here, we focused on recent progress in ABA-mediated stomatal movement and gas exchange, emphasizing its coordination across multiple cell types, rather than guard cells alone. We discussed how spatial/single-cell omics, optogenetics, and AI-assisted analysis could advance ABA research by resolving its cell type signaling events and complex networks. Recent evidence shows that coordinated stomatal and mesophyll functions can buffer CO_2_ diffusion under high temperature and high vapor pressure deficit, stabilizing chloroplast CO_2_ availability and supporting photosynthetic performance (Hu et al 2026). Thus, resolving how ABA coordinates stomatal, mesophyll, and vascular functions is not only important for understanding how plants sustain photosynthesis with minimizing water loss under hot and dry climates, but also a key challenge for future crop resilience and agricultural productivity.

Importantly, this conceptual framework could be extended to other ABA-mediated physiological processes in which cell identities and spatial and dynamic signaling are important. For example, temporal single-cell atlases of *Arabidopsis* seed development and germination by Liew et al (2024) may be leveraged to examine how ABA-associated regulators, transport process, and metabolic states control cell-specific germination programs. Similarly, machine learning–driven analysis of ABA-related defense experimental data predicts how ABA concentration, plant age, and pathogen lifestyle as major factors determine disease-resistance outcomes (Stevens et al 2023). Spatial omics in developing leaves may also help link ABA-regulated different cell states with stomatal development (Liu et al 2020). Together, these studies illustrate how cell-resolution datasets, optogenetic intervention, and AI-assisted modeling can expand ABA research from stomatal control to broader developmental and stress-adaptive signaling networks in the future.

Advance box

- ABA homeostasis is a dynamic and spatial regulated process integrating biosynthesis, catabolism, and reversible conjugation, and is shaped by evolutionary diversification and environmental stress, rather than by operating as a simple linear pathway.
- ABA-mediated stomatal regulation is coordinated by multiple tissues and cell types, including root-to-shoot signaling, bundle sheath cells, mesophyll cells, guard cells, and other cell types.
- Cell type and leaf-surface heterogeneity is evident in stomatal regulation with distinct responses between adaxial and abaxial guard cells, while differential ABA sensitivity and water permeability of spongy and palisade mesophyll cells are also proposed to further contribute to this heterogeneity.
- Single-cell/spatial omics and optogenetics approaches are promising to resolve how ABA-regulated ion transport, signaling, and metabolic modules are integrated across multiple cell types to modulate stomata response.

Outstanding questions:

- How does ABA coordinate ion transport, water movement, and signaling across multiple cell types?
- How does ABA connect ion flux regulation across the plasma membrane and the tonoplast of plant cells?
- What components of ABA biosynthesis are evolutionarily conserved across land plants, and how do they contribute to stomatal regulation in different plant species?
- Can optogenetic manipulation isolate or bypass specific ABA-signaling nodes within individual cell types or simultaneously across multiple interacting cell types in plants?
- Can single-cell/spatial omics, optogenetics, and AI-assisted approach help redesign ABA signaling circuit in plants with smarter water use strategy?

## Data Availability

No new data were generated in support of this review.
